# Assessing large language models as assistive tools in medical consultations for Kawasaki disease

**DOI:** 10.3389/frai.2025.1571503

**Published:** 2025-03-31

**Authors:** Chunyi Yan, Zexi Li, Yongzhou Liang, Shuran Shao, Fan Ma, Nanjun Zhang, Bowen Li, Chuan Wang, Kaiyu Zhou

**Affiliations:** ^1^Department of Pediatric Cardiology, West China Second University Hospital, Sichuan University, Chengdu, China; ^2^Key Laboratory of Birth Defects and Related Diseases of Women and Children (Sichuan University), Ministry of Education, Chengdu, China; ^3^Department of Cardiology, West China Hospital, Sichuan University, Chengdu, China

**Keywords:** artificial intelligence, large language models, medical information, medical education, Kawasaki disease

## Abstract

**Background:**

Kawasaki disease (KD) presents complex clinical challenges in diagnosis, treatment, and long-term management, requiring a comprehensive understanding by both parents and healthcare providers. With advancements in artificial intelligence (AI), large language models (LLMs) have shown promise in supporting medical practice. This study aims to evaluate and compare the appropriateness and comprehensibility of different LLMs in answering clinically relevant questions about KD and assess the impact of different prompting strategies.

**Methods:**

Twenty-five questions were formulated, incorporating three prompting strategies: No prompting (NO), Parent-friendly (PF), and Doctor-level (DL). These questions were input into three LLMs: ChatGPT-4o, Claude 3.5 Sonnet, and Gemini 1.5 Pro. Responses were evaluated based on appropriateness, educational quality, comprehensibility, cautionary statements, references, and potential misinformation, using Information Quality Grade, Global Quality Scale (GQS), Flesch Reading Ease (FRE) score, and word count.

**Results:**

Significant differences were found among the LLMs in terms of response educational quality, accuracy, and comprehensibility (*p* < 0.001). Claude 3.5 provided the highest proportion of completely correct responses (51.1%) and achieved the highest median GQS score (5.0), outperforming GPT-4o (4.0) and Gemini 1.5 (3.0) significantly. Gemini 1.5 achieved the highest FRE score (31.5) and provided highest proportion of responses assessed as comprehensible (80.4%). Prompting strategies significantly affected LLM responses. Claude 3.5 Sonnet with DL prompting had the highest completely correct rate (81.3%), while PF prompting yielded the most acceptable responses (97.3%). Gemini 1.5 Pro showed minimal variation across prompts but excelled in comprehensibility (98.7% under PF prompting).

**Conclusion:**

This study indicates that LLMs have great potential in providing information about KD, but their use requires caution due to quality inconsistencies and misinformation risks. Significant discrepancies existed across LLMs and prompting strategies. Claude 3.5 Sonnet offered the best response quality and accuracy, while Gemini 1.5 Pro excelled in comprehensibility. PF prompting with Claude 3.5 Sonnet is most recommended for parents seeking KD information. As AI evolves, expanding research and refining models is crucial to ensure reliable, high-quality information.

## Background

Kawasaki disease (KD), also known as mucocutaneous lymph node syndrome, is a serious pediatric disease that predominantly affects infants and young children younger than 5 years of age (Wang et al., 2005). Initially, KD manifests with high fever, mucocutaneous inflammation, and cervical lymphadenopathy, which may later progress to involve the coronary arteries and other cardiovascular structures (Kato et al., 1975). KD remains a significant challenge in pediatric healthcare, particularly among East Asian populations. In Japan, the annual incidence of KD reaches approximately 200 cases per 100,000 children, indicating that nearly 1% of children by the age of 4–5 may be affected (Elakabawi et al., 2020; Nakamura et al., 2012). Even in the United States, around 5,440 hospitalizations for KD were reported in 2016 (Iwata et al., 2024). Notably, KD has surpassed rheumatic fever as the primary etiology of acquired cardiac disorders in children across developed nations (Newburger et al., 2016). The severity and complexity of KD’s cardiovascular implications underscore the importance of early detection and intervention. For instance, coronary artery aneurysms (CAA), a critical complication of KD, develop in 25% of untreated cases, but this rate reduces to 4% with timely intervention (McCrindle et al., 2017). Myocardial infarction or rupture of CAA caused by KD can lead to cardiogenic shock or even sudden death. Moreover, as KD patients grow older, the pre-existing cardiac complications often correlate with conditions such as arrhythmias, heart failure, and peripheral arterial occlusion (An et al., 2021). Additionally, even after the acute phase has been controlled, KD patients, particularly those who have previously developed CAA, necessitate lifelong cardiovascular management, as they remain at high risk for coronary thrombosis and stenosis (Kato et al., 1996). Therefore, it is imperative for both parents and physicians to cultivate a comprehensive understanding of Kawasaki disease, from initial diagnosis and treatment to long-term management after recovery.

Currently, the internet has become a dominant platform for accessing health-related information, with a steadily increasing number of individuals utilizing these resources (Daraz et al., 2019). And many patients turn to search engines and online medical content instead of consulting healthcare professionals (Popovac and Roomaney, 2022). In tandem with this digital shift, Artificial intelligence (AI) has established itself as an integral component in the field of medicine (Topol, 2019; Elemento et al., 2021). Recent advancement in AI have catalyzed the development of large language models (LLMs) such as Generative Pre-trained Transformer (GPT) (Sufi, 2024), which shows potential for enhancing medical practice assistance (Haug and Drazen, 2023). Both patients and healthcare providers can access medical information through online AI tools (Hopkins et al., 2023). An increasing number of studies are investigating the potential prospects of LLMs in medicine. These models have been applied in various domains, including diagnostic examination image recognition (Günay et al., 2024; Dehdab et al., 2024), case diagnoses (Lechien et al., 2024) and medical tests (Massey et al., 2023; Ali et al., 2023). With the increasing accessibility and development of LLMs, their application in healthcare has expanded significantly. Prompt engineering, as an emerging field, focuses on the systematic development and refinement of prompts to optimize the performance of LLMs (Wang et al., 2024). The design and quality of prompts can significantly affect the accuracy, relevance, and usefulness of the information produced by these models (Kıyak and Emekli, 2024). More and more individuals may rely on LLMs to seek information regarding health conditions, medications, and medical procedures. Therefore, evaluating the quality and accuracy of LLM-generated content is essential for comprehending information being consumed by patients (Yang et al., 2024).

While existing literature has explored the accuracy and reliability of LLMs in various medical domains, there remains a notable gap in evaluating their performance regarding KD. Given the diverse and complex clinical manifestations of KD, it is imperative to assess the quality of information retrieved using LLMs on this condition. Hence, this research aimed to assess and compare the appropriateness and comprehensibility of different LLMs to clinically pertinent questions related to KD. To evaluate their performance, three distinct prompts were designed to compare the effectiveness of the LLMs under varying conditions.

## Methods

This study was performed during October 2024. To evaluate patient-related information pertinent to KD, a total of 25 questions were carefully formulated through a multi-step process (Table 1). Initially, a preliminary list of 50 questions was generated based on a hospital-wide survey assessing parents’ most frequent inquiries and concerns regarding their children’s KD diagnosis and treatment, and informal interviews with KD patients’ families during follow-up visits. Subsequently, a panel comprising three senior pediatricians, each with over 10 years of experience in general pediatrics and KD management, independently scored these 50 questions and selected 25 highest-scoring questions. The scoring criteria were based on frequency of occurrence and clinical significance. The panel then conducted a series of discussions to refine the question list, ensuring comprehensive coverage of clinically relevant topics. The detailed prompts are provided in Table 2. To minimize any potential bias on the responses, each question was entered in a separate dialogue box.

**Table 1:** Questions concerning Kawasaki disease.

Number	Questions
Q1	What is Kawasaki disease?
Q2	How common is Kawasaki disease in children?
Q3	What are the common symptoms of Kawasaki disease?
Q4	Is Kawasaki disease hereditary?
Q5	What are the types of Kawasaki disease?
Q6	How can I differentiate between typical and atypical Kawasaki disease?
Q7	How is Kawasaki disease diagnosed?
Q8	How is atypical Kawasaki disease diagnosed?
Q9	A child has a high fever. Should I be worried about Kawasaki disease?
Q10	What complications can arise from Kawasaki disease?
Q11	Why does Kawasaki disease cause damage to the heart’s coronary arteries?
Q12	What tests will be used to diagnose Kawasaki disease?
Q13	When should echocardiography be done for Kawasaki patients?
Q14	What are the common treatment options for Kawasaki disease?
Q15	Why is immunoglobulin used to treat Kawasaki disease?
Q16	What role does aspirin play in the treatment of Kawasaki disease?
Q17	When would children need steroids to treat Kawasaki disease?
Q18	Are there any side effects from using steroids to treat Kawasaki disease?
Q19	When would a second dose of immunoglobulin be needed in the treatment of Kawasaki disease?
Q20	How can I help children prevent coronary artery problems caused by Kawasaki disease?
Q21	When can children with Kawasaki disease stop taking aspirin?
Q22	How does the doctor evaluate blood test results for Kawasaki disease?
Q23	Does children with Kawasaki disease need long-term management, and how should it be managed?
Q24	What should we pay special attention to during children’s recovery from Kawasaki disease?
Q25	Will children have long-term effects from Kawasaki disease?

**Table 2:** Prompts applied for each form.

Form name	Prompt provided
No prompting (NO)	(Input the instruction directly)
Parent-friendly prompting (PF)	I am a parent looking to learn more about Kawasaki disease. Please provide answers to the question below in the language that would be appropriate for my understanding. Ensure that your responses are accurate and thorough.
Doctor-level prompting (DL)	I am a doctor attempting to learn the most up-to-date information on Kawasaki disease. Please provide answers to the question below in the language that would be appropriate for my expert-level understanding of medical concepts. Be as specific as possible in your answers.

Each prompt was input before the question.

In this study, we utilized three advanced large language models (LLMs): ChatGPT-4 “Omni” [GPT-4o] (GPT-4, OpenAI, San Francisco, California, United States), Claude 3.5 Sonnet [Claude 3.5] (Claude 3, Anthropic, San Francisco, California, United States), and Gemini 1.5 Pro [Gemini 1.5] (LaMDA, Google, Mountain View, California, United States). The responses generated by these models were systematically evaluated and compared. Each question was posed three times, and the responses were independently reviewed by two experienced physicians, each blinded to the other’s assessments. In case of any discrepancies, a third reviewer was consulted to arbitrate between the two initial assessments. This process ensured the achievement of a unanimous result while maintaining the integrity of the dual-reviewer system. To assess responses on questions concerning KD comprehensively, both subjective ratings and objective evaluations were employed.

Initially, evaluators employed an Information Quality Grade, with the following criteria: Grade 1: completely correct; Grade 2: correct but inadequate; Grade 3: partially correct; Grade 4: entirely incorrect or irrelevant. Then the Global Quality Scale (GQS) was utilized to provide a more detailed evaluation of the response quality (Table 3). Grades 1 and 2 could be collectively referred to as “acceptable responses.” These two information quality assessment tools, previously utilized and validated in prior studies (Wang et al., 2024; Cakir et al., 2024; Dyckhoff-Shen et al., 2024; Onder et al., 2024; Ozgor et al., 2024), demonstrated efficacy in evaluating both the correctness and comprehensiveness of the provided information. Subsequently, responses rated at grade 3 or 4 were subjected to further analysis to categorize the types of errors. These were classified into five distinct categories, (detailed in Table 3). Additionally, the responses were evaluated for different aspects: (Wang et al., 2005) comprehensibility, (Kato et al., 1975) cautionary statements, reference, (Elakabawi et al., 2020) confabulation, as shown in Table 3.

**Table 3:** Procedures evaluating the response quality of LLMs.

Criterion	Description
Information Quality Grade	
Grade 1	Completely correct (Response is entirely accurate and up-to-date with current KD guidelines)
Grade 2	Correct but inadequate (Response is accurate but misses some important aspects of KD)
Grade 3	Partially correct (containing a mixture of accurate and inaccurate information about KD)
Grade 4	Entirely incorrect or irrelevant (Responses directly contradicts the guidelines or offers information irrelevant with KD)
Global Quality Scale (GQS)	
1	Poor quality, poor flow of the site, most information missing, not at all useful for patients
2	Generally poor quality and poor flow, some information listed but many important topics missing, of very limited use to patients
3	Moderate quality, suboptimal flow, some important information is adequately discussed but others poorly discussed, somewhat useful for patients
4	Good quality and generally good flow, most of the relevant information is listed, but some topics not covered, useful for patients
5	Excellent quality and excellent flow, very useful for patients
Error classifications	
A	Misunderstanding of medical terms or jargon
B	Incorrect usage of medical terms
C	Errors in diagnosis/treatment/management
D	Entirely irrelevant information
E	A combination of two or more error types among A–C
Comprehensibility	
Yes/no	Refers to the clarity and ease of understanding for patients
Cautionary statements	
Yes/no	Refers to the presence of a recommendation to consult licensed healthcare professionals or the provision of explicit disclaimers
Reference	
Yes/no	Refers to the presence of any referenced studies in the response
Confabulation	
Yes/no	Refers to the presence of fabricated or distorted information in the response

To quantify readability objectively, we employed two metrics: word count (WoC) and the Flesch Reading Ease (FRE) score. The FRE score, a validated readability measure, assesses text complexity on a 100-point scale, with higher scores indicating greater ease of reading (Jindal and MacDermid, 2017; Hillmann et al., 2023). The interpretation of FRE score is detailed in Table 4. The FRE score is calculated based on two variables: average sentence length (based on the number of words) and average word length (based on the number of syllables) (Jindal and MacDermid, 2017). Both WoC and FRE score were computed by Microsoft Word 365, (Microsoft, Redmond, Washington, United States).

**Table 4:** Interpretation of FRE score.

Score	Readability
91–100	Very easy
81–90	Easy
71–80	Fairly easy
61–70	Standard
51–60	Fairly difficult
31–50	Difficult
0–30	Very difficult

FRE, Flesch Reading Ease.

Statistical analyses were performed by SPSS 26.0 (IBM, New York, NY, United States). Categorical variables, including the Information Quality Grade, were calculated and represented as frequencies and percentages, while continuous variables were represented as median values with interquartile ranges due to their non-normal distribution. Reliability, referred to the repeatability of responses to identical questions, was evaluated using the intraclass correlation coefficient (ICC) of the GQS scores. ICC values were interpreted as follows: excellent reliability (> 0.90), good reliability (0.75–0.90), moderate reliability (0.50–0.75) and poor reliability (< 0.50) (Koo and Li, 2016). Comparative analyses between LLMs and prompt types were conducted using the Kruskal-Wallis test, while categorical data were analyzed via chi-square tests. The Bonferroni correction was applied for post-hoc analysis. All *p* values were two-tailed, with statistical significance set as *p* < 0.05.

## Results

### Evaluation of responses of different LLMs

**Figure 1:**
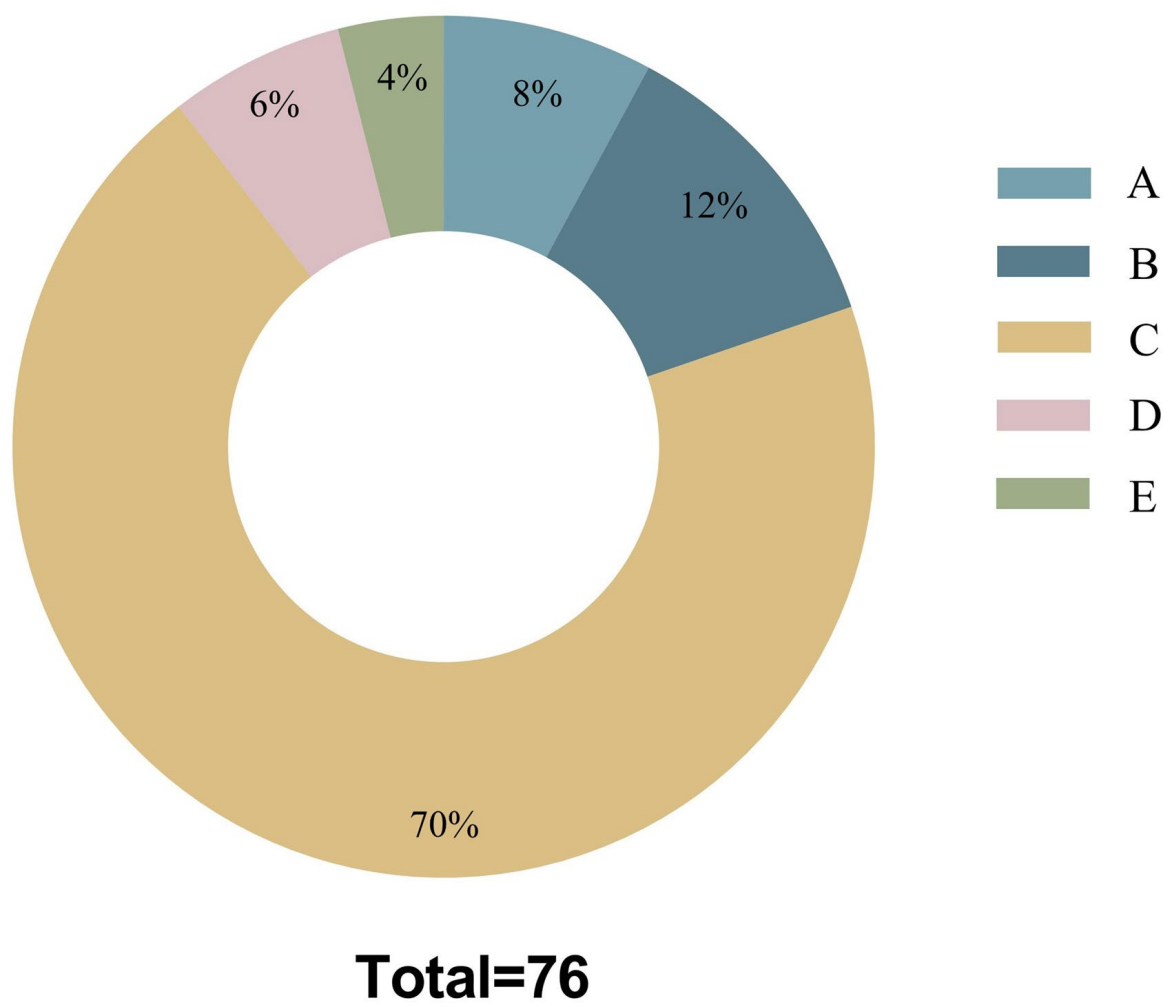
Mistake types among all responses. A: Misunderstanding of medical terms or jargon; B: Incorrect usage of medical terms; C: Errors in diagnosis/treatment/management; D: Entirely irrelevant information; E: A combination of two or more error types among A–C.

There were significant differences in Information Quality Grade, GQS scores, WoC, FRE scores, comprehensibility and cautionary statements among different LLMs (*p* < 0.001). The likelihood of Claude 3.5 giving completely correct responses was 51.1%, which was significantly higher than 18.2% of GPT-4o and 11.1% of Gemini 1.5. Additionally, most of the responses provided by Gemini 1.5 were correct but inadequate (78.2%), significantly higher than GPT-4o (67.1%) and Claude 3.5 (40.4%), with all differences among the three models being statistically significant. There was no significant difference in the frequency of Information Quality Grade 3 or 4 across different LLMs, and it was found that the most prevalent errors were related to diagnosis, treatment, and management (70%) in these responses containing inaccurate or incorrect information (Figure 1). The median GQS values of different LLMs were as follows: Claude 3.5 at 5.0, GPT-4o at 4.0, and Gemini 1.5 at 3.0, with all inter-model comparisons demonstrating statistically significant differences (Table 5).

**Table 5:** Assessment of responses given by three different LLMs.

	ChatGPT-4o	Claude 3.5 Sonnet	Gemini 1.5 Pro	*P*
Information Quality Grade, *n* (%)				
1	41 (18.2)	115 (51.1)^*^	25 (11.1)^‡^	**< 0.001**
2	151 (67.1)	91 (40.4)^*^	176 (78.2)^†‡^	
3	30 (13.3)	18 (8.0)	22 (9.8)	
4	3 (1.4)	1 (0.5)	2 (0.9)	
GQS score	4.0 (3.0–4.0)	5.0 (4.0–5.0)^*^	3.0 (3.0–4.0)^†‡^	**< 0.001**
WoC	231.0 (186.0–267.5)	281.0 (218.5–341.0)^*^	292.0 (222.5–369.5)^†^	**< 0.001**
FRE score	16.0 (0.0–31.3)	19.5 (9.0–31.2)	31.5 (14.2–44.6)^†‡^	**< 0.001**
Comprehensibility, *n* (%)	146 (64.9)	154 (68.4)	181 (80.4)^†‡^	**< 0.001**
Cautionary statements, *n* (%)	80 (35.6)	79 (35.1)	125 (55.6)^†‡^	**< 0.001**
Reference, *n* (%)	0 (0)	0 (0)	225 (100)^†‡^	**< 0.001**
Confabulation, *n* (%)	13 (5.8)	12 (5.3)	9 (4.0)	0.675

GQS, Global Quality Scale; FRE, Flesch Reading Ease; WoC, Word count.

*Claude vs. ChatGPT significantly (*P* < 0.05), ^†^Gemini vs. ChatGPT significantly (*P* < 0.05), ^‡^Gemini vs. Claude significantly (*P* < 0.05). Bold value: *P* < 0.05.

The WoC of responses from GPT-4o (231.0) was significantly lower compared to Claude 3.5 (281.0) and Gemini 1.5 (292.0). Furthermore, in calculating FRE scores, Gemini 1.5 exhibited the highest score (31.5), which was significantly higher than GPT-4o (16.0) and Claude 3.5 (19.5). Both the percentage of responses assessed as comprehensible and the percentage containing cautionary statements from Gemini 1.5 were significantly higher compared to those from GPT-4o and Claude 3.5, with detailed information shown in Table 5. Additionally, Gemini 1.5 consistently referenced studies or websites in all its responses, and the articles or websites cited were verifiable. Conversely, neither GPT-4o nor Claude 3.5 included references in any of their responses.

The ICC values of GQS scores and associated *p*-values for three LLMs responding to three prompts were shown in Supplementary Table 1, where notably, the majority of *p*-values exceeded 0.05, indicating that the observed ICCs may not reflect statistically significant reliability. This outcome may be attributed to insufficient sample sizes within the subgroups. Upon analyzing the reliability of different LLMs, GPT-4o (ICC: 0.429, 95% CI: 0.289–0.565) and Gemini 1.5 (ICC: 0.442, 95% CI: 0.302–0.576) exhibited a poor to moderate reliability, while Claude 3.5 (ICC: 0.334, 95% CI: 0.192–0.480) exhibited a poor reliability (Supplementary Table 2).

### Evaluation of information quality across different LLMs and prompts

**Figure 2:**
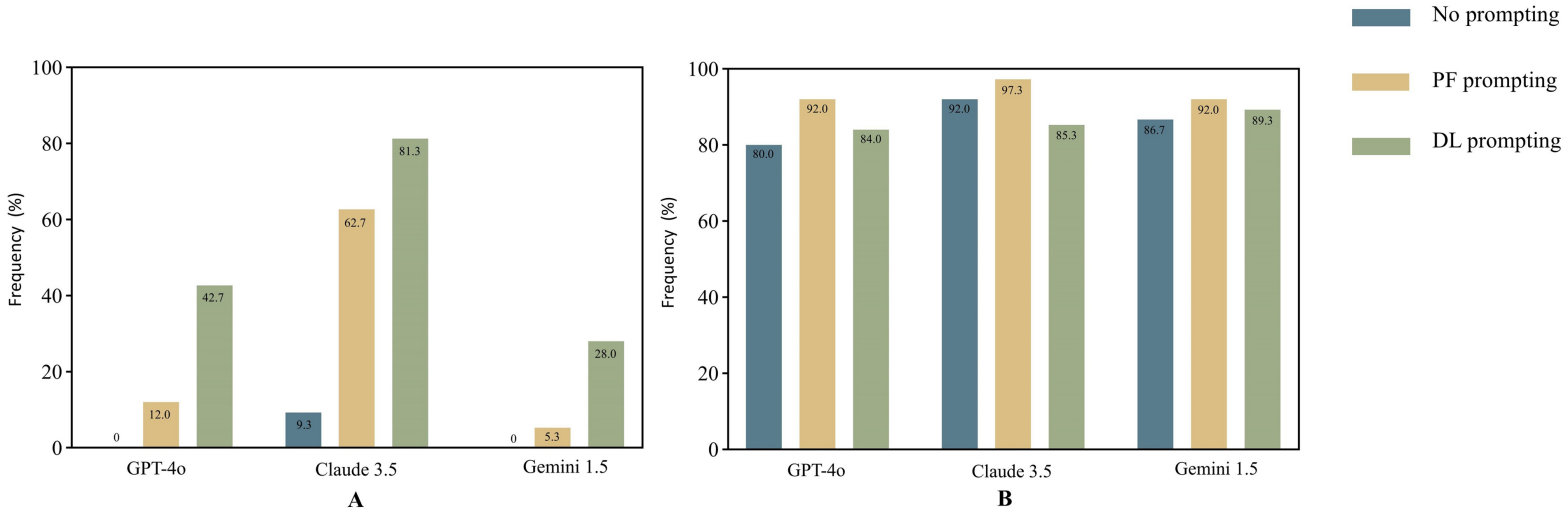
Frequency of Information Quality Grade 1 **(A)** and Grades 1&2 **(B)** across models and prompts.

**Figure 3:**
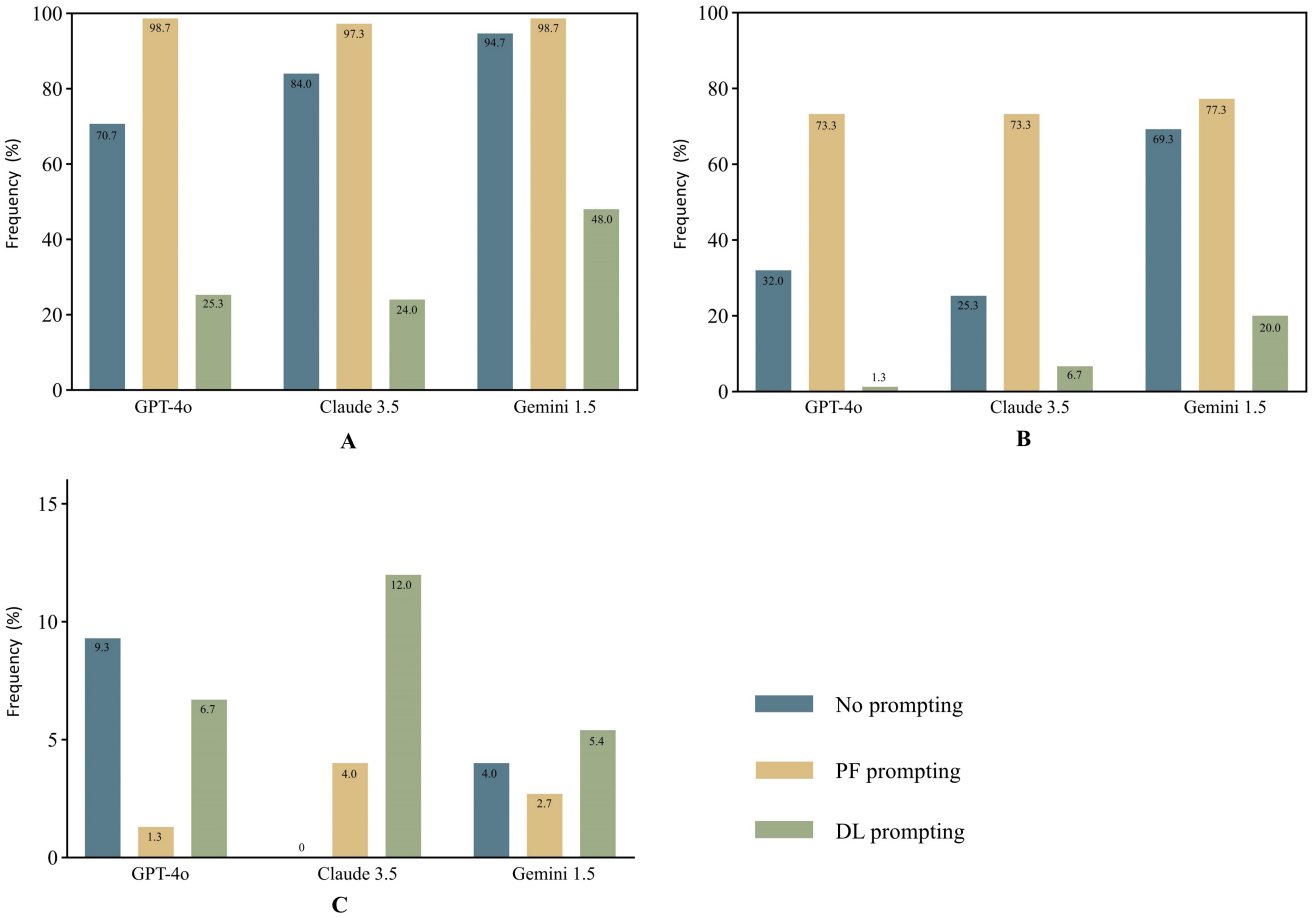
Assessment of comprehensibility **(A)**, cautionary statements **(B)** and confabulation **(C)** across models and prompts.

The findings indicated that Claude 3.5 outperformed the other models in terms of GQS scores and the frequency of achieving Information Quality Grade 1 (Table 5). The proportions of acceptable responses across all models and prompts were considerable, exceeding 80% (Figure 2). The combination of Claude 3.5 and DL achieved the highest GQS score of 5.0 (5.0–5.0) and the highest proportion of completely correct responses at 81.3%, while the combination of Claude 3.5 and PF achieved the highest proportion of acceptable responses at 97.3%. Notably, the combination of Claude 3.5 and DL yielded the highest incidence of confabulation, reaching 12% (Figure 3C), and there was a significant difference observed between DL and NO promoting in Claude 3.5 (Table 6).

**Table 6:** Assessment of responses across different prompts in different LLMs.

	No prompting	Parent-friendly prompting	Doctor-level prompting	*P*
ChatGPT-4o				
Information Quality Grade 1, *n* (%)	0 (0.0)	9 (12.0)^*^	43 (42.7)^†‡^	**< 0.001**
Information Quality Grades 1&2, *n* (%)	60 (80.0)	69 (92.0)	63 (84.0)	0.107
GQS score	3.0 (3.0–3.0)	4.0 (3.0–4.0)^*^	4.0 (4.0–5.0)^†‡^	**< 0.001**
WoC	183 (156–221)	224 (212–258)^*^	267 (227–309)^†‡^	**< 0.001**
FRE score	17.5 (8.5–27.0)	31.9 (26.9–38.8)^*^	0.0 (0.0–2.6)^†‡^	**< 0.001**
Comprehensibility, *n* (%)	53 (70.7)	74 (98.7)^*^	19 (25.3)^†‡^	**< 0.001**
Cautionary statements, *n* (%)	24 (32.0)	55 (73.3)^*^	1 (1.3)^†‡^	**< 0.001**
Confabulation, *n* (%)	7 (9.3)	1 (1.3)	5 (6.7)	0.089
Claude 3.5 Sonnet				
Information Quality Grade 1, *n* (%)	7 (9.3)	47 (62.7)^*^	61 (81.3)^†‡^	**< 0.001**
Information Quality Grades 1&2, *n* (%)	69 (92.0)	73 (97.3)	64 (85.3)^‡^	**0.030**
GQS score	4.0 (3.0–4.0)	5.0 (4.0–5.0)^*^	5.0 (5.0–5.0)^†^	**< 0.001**
WoC	196 (170–239)	281 (248–331)^*^	342 (308–386)^†‡^	**< 0.001**
FRE score	22.7 (15.7–29.3)	33.1 (27.9–38.9)^*^	3.8 (0.0–11.3)^†‡^	**< 0.001**
Comprehensibility, *n* (%)	63 (84.0)	73 (97.3)^*^	18 (24.0)^†‡^	**< 0.001**
Cautionary statements, *n* (%)	19 (25.3)	55 (73.3)^*^	5 (6.7)^†‡^	**< 0.001**
Confabulation, *n* (%)	0 (0.0)	3 (4.0)	9 (12.0)^†^	**0.003**
Gemini 1.5 Pro				
Information Quality Grade 1, *n* (%)	0 (0.0)	4 (5.3)	21 (28.0)^†‡^	**< 0.001**
Information Quality Grades 1&2, *n* (%)	65 (86.7)	69 (92.0)	67 (89.3)	0.571
GQS score	3.0 (3.0–3.0)	3.0 (3.0–3.0)	4.0 (3.0–5.0)^†‡^	**< 0.001**
WoC	271 (216–323)	252 (186–308)	386 (292–430)^†‡^	**< 0.001**
FRE score	31.5 (26.5–43.6)	45.3 (40.4–50.8)^*^	9.8 (3.7–16.1)^†‡^	**< 0.001**
Comprehensibility, *n* (%)	71 (94.7)	74 (98.7)	36 (48.0)^†‡^	**< 0.001**
Cautionary statements, *n* (%)	52 (69.3)	58 (77.3)	15 (20.0)^†‡^	**< 0.001**
Confabulation, *n* (%)	3 (4.0)	2 (2.7)	4 (5.4)	0.646

GQS, Global Quality Scale; FRE, Flesch Reading Ease; WoC, Word count.

*Parent-friendly prompting vs. No prompting significantly (*P* < 0.05), ^†^Doctor-level prompting vs. No prompting significantly (*P* < 0.05), ^‡^Doctor-level prompting vs. Parent-friendly prompting significantly (*P* < 0.05). Bold value: *P* < 0.05.

The proportions of completely correct responses under DL promoting were significantly higher than those under NO and PF prompting across all three models. Conversely, the proportion of acceptable responses under DL prompting was lower than that under PF prompting in these models, with a significant difference observed in Claude 3.5. Furthermore, no significant difference in GQS scores was observed between PF and DL prompting in Claude 3.5. For GPT-4o and Gemini 1.5, the GQS scores under DL promoting were significantly higher than those under PF and NO prompting. For GPT-4o and Claude 3.5, the GQS scores and proportions of completely correct responses under PF promoting were significantly higher than those under NO prompting (Table 6).

### Analysis of the cautionary statements across different LLMs and prompts

The combination of Gemini 1.5 and PF achieved the highest proportion (77.3%) of reminders for users to consult a licensed healthcare professionals or provide of explicit disclaimers in its responses. The proportion of cautionary statements provided under DL promoting was significantly lower than that under NO and PF prompting across all three models, with values of 1.3, 6.7, and 20.0% for GPT-4o, Claude 3.5, and Gemini 1.5, respectively, indicating a notably low level. The proportion of cautionary statements provided under PF promoting was significantly higher than that under NO promoting in GPT-4o and Claude3.5, while in Gemini 1.5, there was no significant difference observed between NO (69.3%) and PF promoting (Table 6). Overall, the percentages of cautionary statements provided by all three models under PF prompting, as well as those from the combination of Gemini 1.5 and NO, were considerable, around 70%.(Figure 3B).

### Evaluation of comprehensibility and readability across different LLMs and prompts

**Figure 4:**
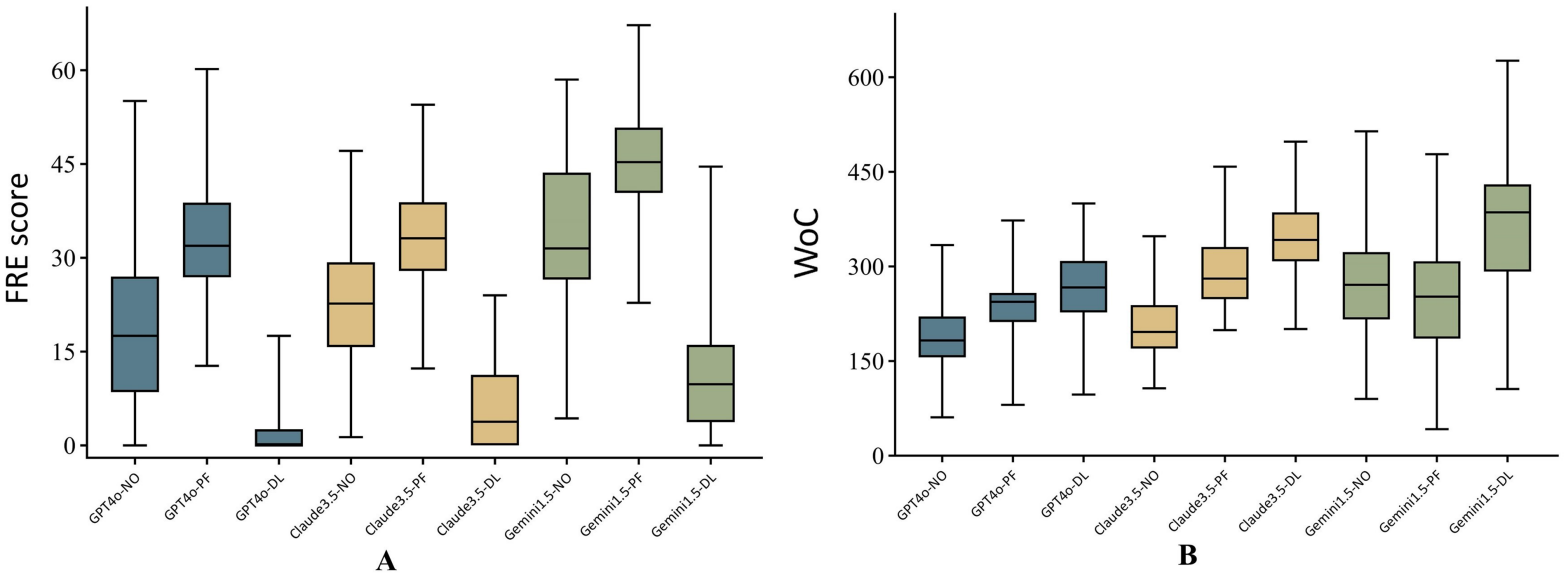
FRE scores **(A)** and word count **(B)** across models and prompts.

The percentages of responses assessed as comprehensible under PF prompting by all three models, as well as those from the combination of Gemini 1.5 and NO, were substantial, exceeding 90%. In contrast, the proportions of responses assessed as comprehensible under DL prompting were significantly lower than those under NO and PF prompting across all three models, with respective values of 25.3, 24.0, and 48.0% for GPT-4o, Claude 3.5, and Gemini 1.5, respectively, suggesting a substantially inadequate level (Figure 3A). The combination of Gemini 1.5 and PF achieved the highest FRE score of 45.3 (40.4–50.8), while the combination of Gemini 1.5 and DL attained the highest WoC of 386 (292–430) (Figure 4). The FRE scores of responses under PF promoting were significantly higher than those under NO and PF prompting across all three model. Detailed analysis of WoC and FRE is presented in Table 6.

## Discussion

Approximately 70–80% of internet users actively seek health-related information online, which plays a crucial role in shaping their initial understandings and perceptions of health issues (Finney Rutten et al., 2019). With the rising prevalence and application of LLMs, there might be an increasing number of individuals depending on these models to obtain details on health conditions, which necessitates healthcare professionals to engage proactively in developing standards and evaluating the quality of information provided by these models (Rajpurkar et al., 2022). Nonetheless, comprehensive data on the utilization of LLMs by healthcare professionals and parents, especially within pediatrics and specifically for KD, remain scarce. This study presents the first evaluation of LLMs’ performance on addressing clinically pertinent questions of KD. Three recently published LLMs, namely Claude 3.5 Sonnet, Gemini 1.5 Pro, and ChatGPT-4o were applied and we also devised two prompts in the perspective of parents and doctors to identify the optimal combination of models and prompting strategy.

Through quantitative scoring approaches including GQS, FRE scores, and additional indices such as Information Quality Grade and WoC, we assessed the responses with an emphasis on educational quality and comprehensibility, while also considering other dimensions. The current results reveals that the responses generated by LLMs: (1) vary in educational quality and proportions in completely correct answers, but generally meet acceptable standards; (2) are mostly easy to understand with varying readability; (3) were unsatisfactory in terms of consistent quality; (4) may exhibit inaccuracies and instances of confabulation occasionally; (5) exhibited notable differences across different LLMs and prompting strategies. Among the 76 inaccurate responses, it was discerned that the most frequent errors pertained to diagnosis, treatment, and management (70%). This prevalence of errors could be attributed to the fact that the majority of inquiries involved KD diagnostics and treatment. Moreover, the variability in treatment protocols across different medical centers may result in discrepancies between evaluators’ assessments and the standards employed by the LLMs.

In the comparative analysis, Claude 3.5 Sonnet demonstrated superior response quality and accuracy, with significantly higher GQS scores (5.0 vs. 4.0 for ChatGPT and 3.0 for Gemini) and a greater proportion of completely correct responses (51.1% vs. 18.2% for ChatGPT and 11.1% for Gemini). This finding aligns with some previous research in the field of LLM application. For instance, Schmidl et al. (2024) reported that Claude 3 Opus demonstrates superior performance in diagnosing head and neck squamous cell carcinoma (HNSCC) compared to ChatGPT 4.0. In contrast, certain studies have suggested a different finding. Meyer et al. (2024) analyzed LLM responses to questions encountered in rhinoplasty practice and found that ChatGPT surpassed both Claude and Gemini in terms of accuracy and overall quality. Additionally, Liu et al. (2024) found no significant difference between the Claude 3 Opus and ChatGPT 4.0 in dermoscopic image analysis for melanoma diagnosis. ChatGPT-4o, on the other hand, provided responses that tended to be more concise, characterized by a lower median word count (231 vs. 281 for Claude and 292 for Gemini).While such brevity can be advantageous in certain contexts, it may result in less comprehensive answers that could omit critical details necessary for fully understanding the topic. Nonetheless, GPT-4o still maintained a reasonable level of accuracy, with GQS scores significantly higher than those of Gemini 1.5 Pro. Meanwhile, in an analysis by Wang et al. (2024) concerning LLM responses on myopia prevention and control, ChatGPT-4.0 exhibited a significantly higher word count than Claude 2 and Gemini, while also achieving the highest scores in comprehensiveness, accuracy, and relevance. These discrepancies may be attributed to various factors, such as differences in studied fields, application scenarios and model versions.

Among three models, Gemini 1.5 Pro excelled in delivering comprehensible responses, as reflected in its higher FRE scores (31.5 vs. 16.0 for ChatGPT and 19.5 for Claude) and percentages of comprehensibility (80.4% vs. 64.9% for ChatGPT and 68.4% for Claude). However, it is important to note that the GQS scores of Gemini were significantly lower than those of the other two models, indicating a potential risk of not delivering high-quality information. This observation is consistent with previous studies (Tepe and Emekli, 2024; Gondode et al., 2024). Despite its lower accuracy, Gemini remains more accessible to a broader patient audience. Notably, each response from Gemini in our study was accompanied by references and external websites that could be independently verified. In some instances, Gemini even cited images from external websites to bolster its textual explanations. These features enhanced the credibility of the information provided, making Gemini suitable for users who prioritize verifiable and easy-to-understand information, such as parents of children with KD. Previous studies had indicated that up to half of all citations presented by ChatGPT were fabricated (Bhattacharyya et al., 2023). And the phenomenon of falsified citations had been extensively documented in the literature (Walters and Wilder, 2023). This highlighted the technological advancements in Gemini, which had effectively mitigated this issue. Additionally, Gemini exhibited a higher proportion of responses containing cautionary statements, which recommended consulting professionals or included explicit disclaimers. While these statements could encourage parents to be more vigilant and nuanced in evaluating the information, they might also imply a lack of confidence in the medical judgments provided. Therefore, further investigations are warranted to assess the potential risks and benefits of this response mode (Wang et al., 2024).

An appropriate prompt could improve the accuracy and comprehensibility of responses to medical questions and different prompts had variable effects across diverse models (Wang et al., 2024). In our study, we found that DL prompting consistently outperformed NO prompting in terms of overall educational quality. However, from assessment of comprehensibility and readability, the responses generated under DL prompting were often more challenging for users to understand. Furthermore, within the Claude 3.5, there was a significantly higher incidence of confabulation with DL prompting compared to NO prompting, and the proportion of acceptable responses under DL prompting was significantly lower than that observed with PF prompting. These findings illustrated that while DL prompting could provide high-quality and comprehensive information, it may also carry a higher likelihood of inaccuracies in certain scenarios. Nonetheless, recommending the use of DL prompting for medical professionals might still prove beneficial, as it offered deeper insights, and medical professionals were more likely to critically evaluate the information provided. For ChatGPT-4o and Claude 3.5 Sonnet, the responses generated under PF prompting demonstrated significantly improvements in educational quality, accuracy, and comprehensibility compared to those generated using NO prompting, which indicated that PF prompting not only enhanced the quality, but also facilitated better user understanding. Moreover, the performance of Gemini 1.5 Pro did not exhibit significant differences in response quality and comprehensibility between PF and NO prompting. However, PF prompting within Gemini did result in superior FRE scores and interestingly, incorporated the use of metaphors in certain responses, which could aid readability and make the information more accessible for patients.

Among the three models, Claude 3.5 Sonnet demonstrated the highest overall educational quality. The quality achieved under PF prompting within Claude was comparable to that of DL prompting, both of which were statistically superior to NO prompting. Furthermore, the proportion of acceptable responses generated by the combination of Claude 3.5 and PF prompting approached nearly 100%, indicating a minimal likelihood of inaccuracies in the responses. This combination also exhibited considerable comprehensibility, with the percentage of comprehensible responses nearing 100%. Consequently, for parents seeking information on KD, Claude with PF prompting emerges as the optimal choice due to its outstanding educational quality, accuracy and clarity.

While this was the first study assessing the responses of LLMs concerning KD, it had several limitations. Firstly, since a LLM is trained until a specific cut-off date, the version utilized in this analysis may have been updated subsequently, potentially leading to variations in responses in future iterations. Secondly, although the questions were meticulously devised through a multi-step process, their limited number may not fully capture the comprehensive scope of the topic. Despite the implementation of a double-review process, the assessment remained subjective, causing unavoidable confounders in our evaluation. Additionally, as the evaluations of comprehensibility were performed by pediatric professionals rather than KD patients or parents of children with KD, the results s may be subject to biases and not accurately reflect a patient’s perspective. Furthermore, the investigation was conducted in English, which is the predominant global language. Future research could explore the responses of LLMs in similarly widely used languages, such as Chinese. Moreover, although cautionary statements promoted responsible and prudent application, the exploration of the potential advantages and drawbacks inherent in this approach remains necessary.

In future research, broadening the scope and depth of inquiry and assessment, would provide a more comprehensive understanding of LLMs’ capabilities and limitations in pediatric and KD contexts. Considering clinical applications of LLMs, particularly from the parents’ perspective, KD is a challenging disease that requires long-term management. Therefore, prompt engineering could play a crucial role in guiding patients to inquiry medical questions correctly, potentially improving patient education and effectively addressing their queries. Further research is warranted to refine prompt engineering across LLMs, tailoring them to specific medical inquiries and target audiences. Furthermore, it is essential to develop specialized LLMs with advanced medical expertise to assist physicians in the field such as KD diagnosis, cardiac ultrasound interpretation, and decision-making (Pan and Jiao, 2024). As AI technology evolves, continuous evaluations and updates would be necessary to ensure that these tools remain reliable and effective in clinical application.

## Conclusion

The utilization of LLMs for patient education on KD holds substantial potential as a help resource. Generally, responses generated by different LLMs meet to acceptable standards and are mostly comprehensible, albeit with variations in readability and educational quality. However, the consistency in quality remains unsatisfactory and issues of misinformation and confabulation persist. Therefore, parents and physicians should be cautious when utilizing LLMs for medical information on KD. Notable discrepancies were observed across different LLMs and prompting strategies. Claude 3.5 Sonnet demonstrated superior response quality and accuracy, whereas the Gemini 1.5 Pro excelled in delivering comprehensible responses. The efficacy of different prompts varied across the various models, with Claude 3.5 Sonnet employing PF prompting being most recommended for parents seeking information on KD. As AI technology rapidly advances, it is crucial to broaden the scope and depth of inquiry, continuously evaluate and update models, and develop specialized LLMs with advanced medical expertise to assist physicians.

## Data Availability

The raw data supporting the conclusions of this article will be made available by the authors, without undue reservation.
