# Supplementary material for: Assessing large language models as assistive tools in medical consultations for Kawasaki disease
Source: Front Artif Intell. 2025 Mar 31;8:1571503. doi: 10.3389/frai.2025.1571503 (PMC11994668; doi:10.3389/frai.2025.1571503)
Supplement: Supplementary file 1 [file Table_1.DOCX]

| **Supplementary Table 1**: The ICC values of GQS scores for different LLMs responding to 3 prompts | | | | |
| --- | --- | --- | --- | --- |
| Model | Prompt | ICC | *P* | 95% CI |
| ChatGPT-4o | NO | 0.145 | 0.109 | -0.077-0.420 |
|  | PF | 0.306 | **0.006** | 0.067-0.563 |
|  | DL | 0.022 | 0.410 | -0.168-0.287 |
| Claude 3.5 Sonnet | NO | 0.033 | 0.378 | -0,172-0.310 |
|  | PF | 0.233 | 0.030 | -0.007-0.504 |
|  | DL | -0.078 | 0.744 | -0.242-0.173 |
| Gemini 1.5 Pro | NO | 0.064 | 0.255 | -0.112-0.314 |
|  | PF | 0.498 | **< 0.001** | 0.259-0.712 |
|  | DL | -0.240 | 0.985 | -0.362- -0.028 |
| PF Parent-friendly, DL Doctor-level | | | | |

| **Supplementary Table 2**:The ICC values of GQS scores of different LLMs | | | |
| --- | --- | --- | --- |
| Model | ICC | 95% CI | *P* |
| ChatGPT-4o | 0.429 | 0.289-0.565 | **< 0.001** |
| Claude 3.5 Sonnet | 0.334 | 0.192-0.480 | **< 0.001** |
| Gemini 1.5 Pro | 0.442 | 0.302-0.576 | **< 0.001** |
